# Association between basilar artery configuration and Vessel Wall features: a prospective high-resolution magnetic resonance imaging study

**DOI:** 10.1186/s12880-019-0388-3

**Published:** 2019-12-26

**Authors:** Ziqi Xu, Mingyao Li, Zhikai Hou, Jinhao Lyu, Na Zhang, Xin Lou, Zhongrong Miao, Ning Ma

**Affiliations:** 1 Department of Neurology, The First Affiliated Hospital of College of Medicine, Zhejiang University, Hangzhou, China; 2 Department of Interventional, Neuroradiology, Beijing Tiantan Hospital, Capital Medical University, China National Clinical Research Center for Neurological Diseases, Center of Stroke, Beijing Institute for Brain Disorders, Beijing, China; 3 Paul C. Lauterbur Research Center for Biomedical Imaging, Shenzhen Institutes of Advanced Technology, Chinese Academy of Sciences, Shenzhen, China; 4 Department of Radiology, Chinese PLA General Hospital, Beijing, China

**Keywords:** Vertebrobasilar artery, Atherosclerotic disease, High-resolution MRI, Collateral circulation, Anatomical configuration

## Abstract

**Background:**

The relationship between intracranial vessel configuration and wall features remains poorly investigated. Therefore, we aimed to investigate the relationship between the distal and proximal anatomical configuration of basilar artery (BA) and BA vessel wall features on high-resolution magnetic resonance imaging (HRMRI).

**Methods:**

From September 2014 to January 2017, patients with suspected symptomatic intracranial arterial stenosis underwent HRMRI. Patients with severe BA stenosis were selected for this prospective study and divided into two groups corresponding to complete and incomplete BA configuration based on characteristics of the bilateral vertebral arteries and posterior cerebral arteries. Culprit blood vessel wall features on HRMRI included plaque enhancement, intraplaque hemorrhage, remodeling patterns, and plaque distribution. Culprit vessel wall features were compared between patients in the complete and incomplete BA configuration groups.

**Results:**

Among the 298 consecutively enrolled patients, 34 had severe BA stenosis. Twenty patients had complete anatomical BA configuration and another 14 of them displayed incomplete configuration. There were no significant differences in vessel wall features between the complete and incomplete configuration patient groups. However, the proximal configuration of BA was associated with intraplaque hemorrhage (*p* = 0.002) while the distal configuration of BA correlated with strong enhancement of BA plaque (*p* = 0.041).

**Conclusions:**

No association was found between the complete and incomplete BA configuration groups and blood vessel wall features. The proximal configuration of BA was related with intraplaque hemorrhage and the distal configuration of BA was associated with strong plaque enhancement. Further studies are warranted to confirm these findings.

**Trial registration:**

URL: Unique identifier: NCT02705599 (March 10, 2016).

## Background

Basilar artery (BA) atherosclerotic occlusive disease is the most common cause for posterior circulation strokes, with potentially disastrous patient outcome and a high risk of recurrent stroke [1, 2]. Anatomically, BA arises from the junction of the bilateral vertebral arteries (VAs) and then divides into the bilateral posterior cerebral arteries (PCAs) and branches out into two pairs of cerebellar arteries. VA diameter may vary within the same individual, with only 6–26% of cases exhibiting equal size bilaterally in angiographic studies [3]. Previous studies identified VA hypoplasia as a predisposing factor for posterior circulation stroke [4, 5]. Conversely, VA dominance was shown to often cause BA curvature and the subsequent development of peri-vertebrobasilar junction infarcts [4, 5]. In other studies, fetal type PCA (fPCA) was shown to be a predisposing factor to ischemic events in the posterior circulation [6, 7].

The grade of intracranial artery stenosis and vulnerability of plaques are used as indications to guide the clinical management of patients [8]. One study demonstrated that symptomatic stenosis of the vertebrobasilar arteries was associated with a greatly increased risk of recurrent stroke [9]. Other studies demonstrated that vulnerability of intracranial artery plaque and cerebral hypoperfusion at the distal stenotic site were highly associated with stroke events and recurrence [10, 11]. Furthermore, an anterior circulation acute ischemic stroke study revealed associations between collateral circulation and thrombus characteristics, with patients displaying higher collateral scores having a lower thrombus burden and larger number of previous thrombi [12, 13].

However, the relationship between intracranial vessel configuration and wall features remains poorly investigated. BA configuration plays a role in posterior circulation hemodynamics and may influence the vessel wall features of BA. To test this hypothesis, we investigated the demographics, variants of VA and PCA, and BA vessel wall features. We compared the relationship between different BA configurations and vessel wall features using HRMRI.

## Methods

This is a prospective registry study that has been approved by the ethics committee of our hospital. Written informed consent was obtained from the patients or their relatives.

### Enrollment of patients

Patients suspected to have symptomatic intracranial atherosclerotic stenosis (ICAS) at admission were enrolled. All patients received thorough medical evaluations to determine the underlying cause using a range of techniques including carotid artery duplex scan, transcranial Doppler, echocardiography, electrocardiography, computer tomography (CT), magnetic resonance imaging (MRI), CT angiography (CTA), magnetic resonance angiography (MRA), and digital subtraction angiography (DSA). Patients were included in the BA study according to the following criteria: 1) age ≥ 18 years; 2) ischemic stroke or transient ischemic attack (TIA) in the BA regions within 90 days following enrollment; 3) basilar artery stenosis ≥70% and without coexistent ≥50% ipsilateral extracranial vertebral artery stenosis; 4) absence of potential sources of cardioaortic embolism based on the modified Trial of ORG 10172 in Acute Stroke Treatment (TOAST) classification [14]; 5) one or more risk factors for atherosclerosis; 6) all the patients received DSA examination. Other risk factors were recorded for comorbidities including hypertension, dyslipidemia, diabetes, smoking, and obesity.

Patients with the following conditions were excluded: 1) non-atherosclerotic cerebral vasculopathies such as vasculitis and artery dissection, diagnosed by comprehensive laboratory examinations (such as erythrocyte sedimentation rate or C-reactive protein elevation, antinuclear antibody, or antiphospholipid antibody positivity), vascular imaging, and clinical evaluation. 2) contraindication to MR examination, instable clinical state precluding MR examination.

### HRMRI acquisition and analysis

All HRMRI studies were performed on a DISCOVERY MR750 3.0 T (GE Healthcare, Waukesha, WI, USA) or a 3 T Trio MRI scanner (Siemens Healthcare, Ehrlangen, Germany). More details can be found in the study protocol (see Additional file 1: Table S1). Image reconstruction was conducted using the Reformate tool in Advantage Workstation 4.5 (GE Healthcare) and 3D multiple planer reconstruction tool in Siemens workstation. MR images were then processed for all the identified plaques using commercially available software (VesselMass; Leiden University Medical Center, Leiden, The Netherlands).

A culprit plaque was defined as the single lesion present in the artery supplying the infarct zone, or as the most severe stenotic lesion when multiple plaques were present in the supplying artery [15, 16].

The arterial remodeling index (RI) was calculated as the ratio of outer wall area (OWA) at the site of maximal lumen narrowing to that at the reference site (RI=OWA lesion/OWA reference) [17]. The reference site was selected based on the Warfarin-Aspirin Symptomatic Intracranial Disease (WASID) trial method [18]. Three remodeling categories have previously been described, with RI ≥1.05 defined as positive remodeling, 0.95 < RI < 1.05 as intermediate remodeling, and RI ≤0.95 as negative remodeling. Plaque distributions were dichotomized into diffuse and non-diffuse patterns at culprit lesion. The anatomical location of the plaque was recorded as ventral, dorsal, left, and right quadrants [19]. Plaques spreading across four quadrants were defined as diffuse and that involving ≤3 quadrants were defined as non-diffuse. Intraplaque hemorrhage (IPH) was defined as a signal intensity greater than 150% of T1 signal of adjacent muscle [20]. As for plaque enhancement, non-enhancement was defined as similar to, or less than, that of normal intracranial arterial walls nearby, while enhancement meant signal intensity greater than non-enhancement and less than, or greater than, that of the pituitary infundibulum [15].

We adopted the same principle when interpreting HRMRI imaging for arterial remodeling and vessel wall features as we published before with small intra-observer and inter-observer variability [10, 17, 21]. The intra- and inter-observer variability of the two HRMRI scanners and identified vessel wall features were good to excellent (weighted k = 0.82, 95% CI: 0.46, 1.00 and 0.83, 95% CI: 0.41–1.00, respectively).

### Definition of BA configurations

**Fig. 1:**
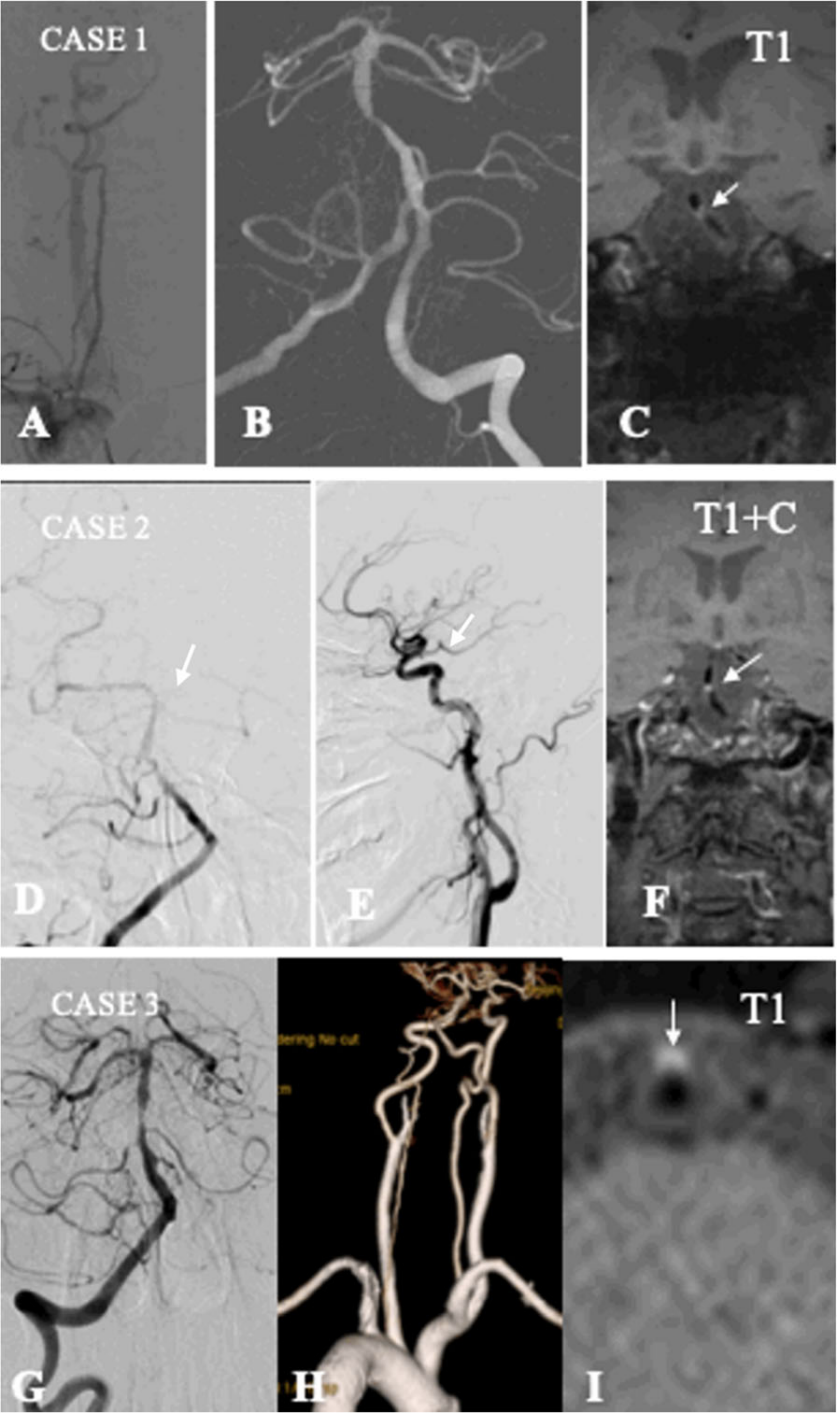
Example of complete and incomplete artery configuration of basilar artery. Case 1: the complete configuration of BA. **a**: the normal right VA with severe tortuosity of the first segment; **b**: normal left VA with severe stenosis of BA; **c**: HRMRI showing intraplaque hemorrhage of BA plaque on T1 weight imaging. Case 2: the incomplete distal configuration of BA. **d**: right VA angiography showing severe stenosis of BA without left PCA. **e**: left common carotid artery angiography showing fetal type PCA. **f**: HRMRI showing strong enhancement of BA plaque. Case 3: the incomplete proximal configuration of BA. G: dominant VA angiography showing severe stenosis of BA. H: CT angiography showing right VA hypoplasia. I: HRMRI showing intraplaque hemorrhage of BA plaque on T1 weight imaging

BA anatomy was defined as having an either complete or incomplete configuration (see Fig. 1). The patients with normal bilateral vertebral arteries and posterior cerebral arteries were categorized as presenting complete BA configuration (see Fig. 1a-c). Patients with fPCA (see Fig. 1d-f) and/or dysplasia in one VA (see Fig. 1g-i) were identified by DSA and/or CTA, MRA as having incomplete BA configuration. The presence of posterior communicating arteries was also recorded. Hypoplasia was defined as VA having a diameter < 2 mm, ending in the posterior inferior cerebellar artery (PICA), or having a lumen diameter more than 50% difference [4, 5]. fPCA is a common anatomic variation that was defined as a posterior cerebral artery originating from the internal carotid artery, in the absence of the P1 segment of PCA or presence of PCA P1 segment dysplasia [6, 7]. Two neurologists (Z.Q.X. and N.M) reviewed the DSA images independently and discrepancies were resolved by consensus.

### Statistical analysis

Continuous variables were presented as means ± SD or median with interquartile range. Categorical variables were presented as percentages. All baseline characteristics, plaque enhancement, intraplaque hemorrhage, arterial remodeling patterns, and plaque distribution were compared with χ2 test for categorical variables and one-way analysis of variance or the Kruskal-Wallis test for continuous variables between the complete and incomplete configuration groups. The analyses were performed using SPSS 23.0 statistical software (IBM, Chicago, IL, USA). A two-tailed *p* value less than 0.05 was considered statistically significant.

## Results

### Baseline characteristics

**Fig. 2:**
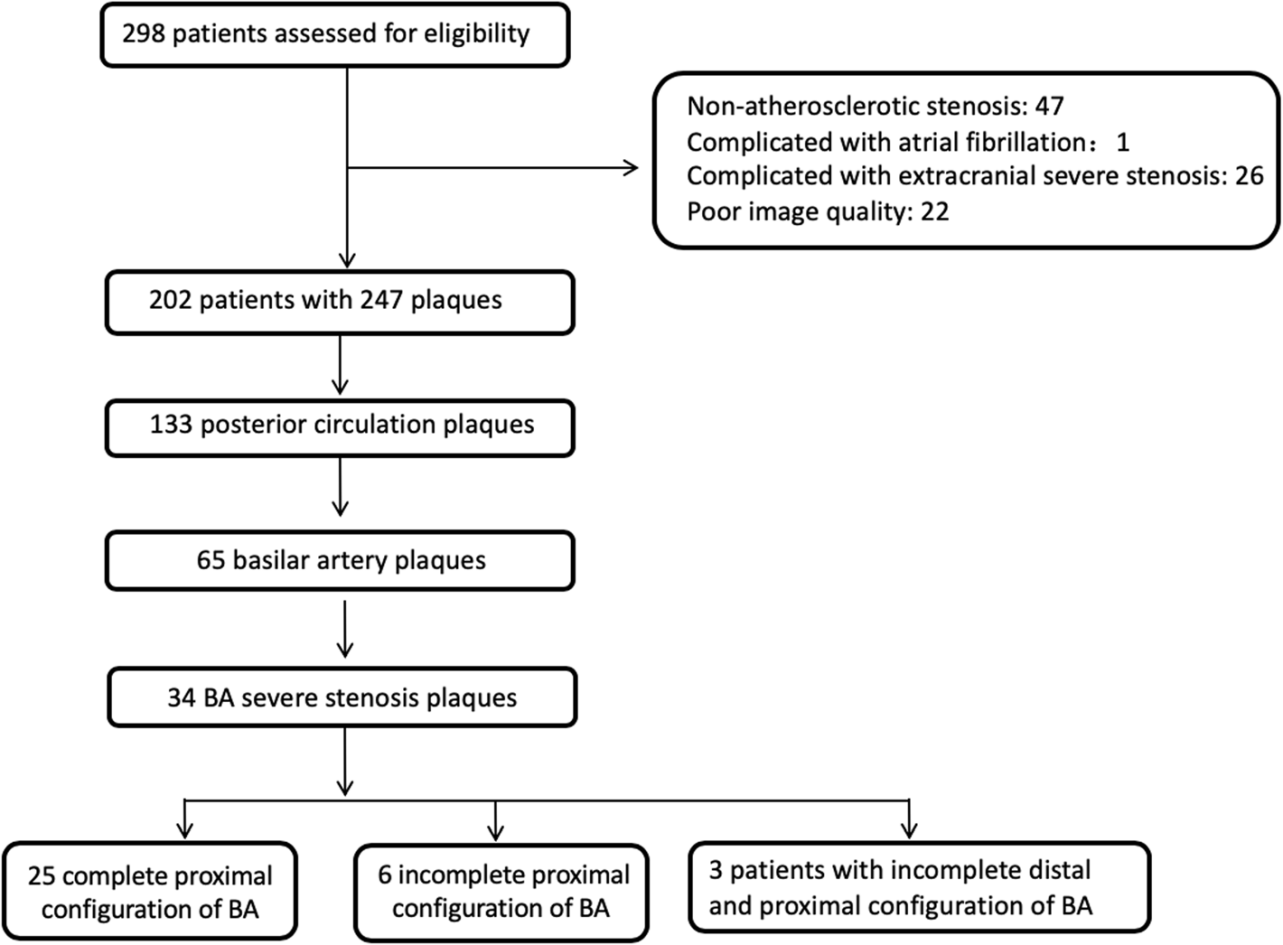
Flow chart of study

From September 2014 to January 2017, among 298 consecutively enrolled patients, 34 patients were included in our study (see Fig. 2). Among them, 6 patients had a TIA and 28 a stroke. Among stroke patients, 1 was hemodynamic mechanism, 12 patients were perforator mechanism, 6 were embolic mechanism and 9 patients were mixing mechanism. The mean time from events to HRMRI examination was 37.75 ± 25.84 days. 64.2% of all the identified plaques showed enhancement; 20.6% were accompanied by intraplaque hemorrhage; 50.0% displayed positive remodeling; and 97.1% had diffuse distribution. The demographic data and risk factors between the complete and incomplete configuration groups were not statistically different (For detailed results, see Table 1).

**Table 1:** Demographic and clinical characteristics of patients in the complete and incomplete basilar artery configuration groups

Variable	All patients (*n* = 34)	Complete configuration of BA (*n* = 20)	Incomplete configuration of BA (*n* = 14)	*P* value
Age-years (SD)	58.77 ± 9.39	59.18 ± 8.13	58.35 ± 10.74	0.803
Body mass index-kg/m^2^(SD)	26.60 ± 2.83	26.24 ± 2.05	26.96 ± 3.27	0.463
LDL-mmol/L (SD)	1.91 ± 0.71	1.77 ± 0.76	2.05 ± 0.65	0.263
Male-No. (%)	28 (82.4)	16 (94.1)	12 (70.6)	0.072
Hypertension-No. (%)	28 (82.4)	12 (70.6)	16 (94.1)	0.072
diabetes-No. (%)	14 (41.2)	7 (41.2)	7 (41.2)	1.000
dyslipidemia-No. (%)	13 (38.2)	3 (17.6)	10 (58.8)	0.013
smoking-No. (%)	25 (73.5)	12 (70.6)	13 (76.5)	0.697
Coronary artery disease-No. (%)	8 (23.5)	5 (29.4)	3 (17.5)	0.419
Qualifying events-No. (%)				
TIA	6 (17.6)	5 (29.4)	1 (5.9)	0.072
infarction	28 (82.4)	12 (70.6)	16 (94.1)	0.072
Time from event to HRMRI-days (IQR)	33 (16–52)	33 (13–52)	33 (23–46)	0.462
Enhancement grade^a^-No. (%)				
None	10 (35.7)	7 (70.0)	3 (30.0)	0.453
Mild to moderate	8 (28.6)	5 (62.5)	3 (37.5)	0.903
Strong	10 (35.7)	5 (50.0)	5 (50.0)	0.387
Intraplaque hemorrhage-No. (%)	7 (20.6)	2 (28.6)	5 (71.4)	0.068
Arterial remodeling-No. (%)				
Negative	12 (35.3)	11 (64.7)	6 (35.3)	0.486
Positive	17 (50.0)	9 (52.9)	8 (47.1)	
Distribution patterns-No. (%)				
Non-diffuse	1 (2.9)	0 (0)	1 (100.0)	0.225
Diffuse	33 (97.1)	20 (60.6)	13 (39.4)	

^a^ Data from 28 patients

### The association between BA configuration and vessel wall features

Among 34 patients with culprit severe BA stenosis, 20 patients (58.8%) presented a complete BA configuration and 14 patients (41.2%) an incomplete configuration. There were no statistical difference in vessel wall features between the complete and the incomplete configuration groups (For detailed results, see Table 1).

Furthermore, 8 patients (23.5%) had fPCA, including 2 patients (5.9%) with bilateral fPCA. Eleven patients (32.4%) had hypoplastic VA, including 3 patients with a VA (8.8%) ending in a PICA and 7 patients (20.6%) with VA occlusion. The proximal configuration of BA was associated with intraplaque hemorrhage (*p* = 0.002) and the distal configuration of BA correlated with strong enhancement of BA plaque (*p* = 0.041) (For detailed results, see Tables 2 and 3).

**Table 2:** Comparison of plaque features between complete and incomplete basilar artery proximal configuration

Characteristics	Complete proximal configuration (*n* = 25)	Incomplete proximal configuration (*n* = 9)	*P* value
Enhancement grade^a^-No. (%)			
None	8 (80.0)	2 (20.0)	0.454
Mild to moderate	5 (62.5)	3 (33.3)	0.508
Strong	7 (70.0)	3 (30.0)	0.901
Intraplaque hemorrhage	2 (28.6)	5 (71.4)	**0.002**
Remodeling patterns-No. (%)			
Negative	8 (66.7)	4 (33.3)	0.503
Positive	13 (76.5)	4 (23.5)	0.697
Distribution patterns-No. (%)			
non-diffuse	0 (0)	1 (100)	0.274
diffuse	25 (75.8)	8 (24.2)	0.091

^a^Data from 28 patients

Significant differences are highlighted in bold

**Table 3:** Comparison of plaque features between patients with complete and incomplete distal basilar artery configuration

Characteristics	Complete distal configuration (*n* = 26)	Incomplete distal configuration (*n* = 8)	*P* value
Enhancement grade^a^-No. (%)			
None	9 (90.0)	1 (10.0)	0.272
Mild to moderate	7 (87.5)	1 (12.5)	0.466
Strong	6 (60.0)	4 (40.0)	**0.041**
Intraplaque hemorrhage	5 (71.4)	2 (28.6)	0.724
Remodeling patterns-No. (%)			
Negative	10 (83.3)	2 (16.7)	0.486
Positive	11 (64.7)	6 (35.3)	0.106
Distribution patterns-No. (%)			
Non-diffuse	0 (0)	1 (100)	0.067
diffuse	26 (78.8)	7 (21.2)	

^a^Data from 28 patients

Significant differences are highlighted in bold

## Discussion

When the BA becomes the site of an atherosclerotic lesion, whether cerebral vascular configuration is associated with the vessel features remains unclear. Hence, we aimed to explore the effects of varying anatomical configurations on culprit plaque characteristics in BA. To our knowledge, this is the first study to explore the correlation of posterior circulation artery configuration with culprit plaque features in BA using 3D HRMRI.

The present study found no statistical differences in vessel wall features between the complete and incomplete configuration of BA. Conversely, variations of configuration BA tree had no relationship with the vessel wall features of BA. The fPCA and VA lumen size difference are common variations of posterior circulation. fPCA is a common anatomical variant of the circle of Willis and can be accompanied by hypoplastic BA [22]. In our study, 23.5% of patients had fPCA, consistent with previous reports [22]. Lochner et al. found that fPCA accompanied by hypoplastic BA may predispose individuals to ischemic events in the posterior circulation [6]. Another study found that unequal VA diameter may cause BA curvature and the subsequent development of peri-vertebrobasilar junctional infarcts [5]. Ravensbergen et al. showed that the geometry of the vertebrobasilar junction correlated with occurrence of atherosclerotic plaque at the apex of the vertebrobasilar junction and lateral wall of BA [23]. In our study, there were 10 patients (29.4%) with non-dominant VA occlusion, and among them, 3 patients’ VA (8.8%) ended in a PICA. Overall, although variants of VA and PCA were associated with ischemic stroke, variations in the vertebrobasilar tree configuration had no relationship with BA vessel wall features. The results suggest that variants of artery configuration cannot trigger the formation of plaques.

Our study showed that the proximal configuration tree of BA was associated with strong plaque enhancement. In the present study, 64.2% of plaques showed enhancement. A previous study found that BA plaque enhancement and composition correlated with stroke events [24]. Plaque enhancement reflects the extent of vessel wall inflammation. The incomplete configuration of proximal VA may cause differences in long-term patient outcomes, but further studies are warranted to confirm this hypothesis.

Our study also showed that the proximal configuration tree of BA was associated with strong plaque enhancement and the distal configuration tree of BA was closely related with intraplaque hemorrhage. In the present study, 20.6% presented intraplaque hemorrhage on HRMRI. Plaque enhancement and intraplaque hemorrhage on HRMRI are markers of plaque destabilization and progression strongly associated with stroke events [24], which are also associated with endothelial dysfunction and neovascularization of the artery wall [25, 26]. The relationship between incomplete distal configuration of BA and intraplaque hemorrhage is difficult to explain given our present understanding. The incomplete distal configuration of BA indicated different hemodynamics and blood flow reserve on the top of BA. The fPCA indicated that most or all the blood flow of PCA was from the ipsilateral internal carotid artery. In a complete BA tree, the most common distribution pattern of blood flow at the top of the BA is as a parallel arrangement of vessels [27]. The fPCA changes this flow pattern, thereby affecting the regional vessel wall shear force. A study showed that blood flow shear stress acts on vessel walls, causing endothelial injury and plaque instability and presenting with plaque enhancement [28]. Poor collateral circulation and high grade of stenosis produced high-speed blood flow around the plaque, leading to erosion of the plaque’s fibrous cap or endothelium injury in the middle cerebral artery and carotid artery and subsequently causing plaque enhancement [29, 30]. The underlying mechanism of plaque enhancement correlated with distal incomplete configuration of BA needs to be further elucidated.

Unequal VA diameter was found to cause BA curvature and development of peri-vertebrobasilar junctional infarcts [5]. Dominant VA flow acted on the contralateral wall of BA to generate the tortuous geometry of BA, which strongly affected velocity and wall shear stress distribution [31], and finally, triggered the formation of BA plaque and affected intraplaque hemorrhage. The geometry of vertebrobasilar artery was shown to be correlated with occurrence of atherosclerotic plaques and plaque distribution [31]. Asymmetric VAs causing BA bending is a chronic process and is not only influenced by shear stress but also by vascular risk factors [31, 32]. The mechanism underlying the correlation between intraplaque hemorrhage and incomplete proximal configuration of BA also needs to be further elucidated.

This study has some limitations. First, the number of subjects is rather small and all patients come from a single stroke center, meaning that selection bias may be a concern. Second, our study population exclusively represented a high degree of stenosis, meaning that caution should be exerted when generalizing the findings.

## Conclusions

We found that the complete configuration of BA was not associated with BA vessel features; the proximal configuration of BA was related to intraplaque hemorrhage and the distal configuration of BA was associated with strong plaque enhancement. Further studies are warranted to confirm the findings.

## Supplementary information

**Additional file 1: Tables S1.** Parameters of multiple sequences on GE and Siemens MR scanners.

## Data Availability

The datasets used and analysed during the current study are available from the corresponding author on reasonable request.

## Abbreviations

BA: Basilar artery
CT: Computer tomography
CTA: CT angiography
DSA: Digital subtraction angiography
HRMRI: High-resolution magnetic resonance imaging
ICAS: Intracranial atherosclerotic stenosis
IPH: Intraplaque hemorrhage
MRA: Magnetic resonance angiography
MRI: Magnetic resonance imaging
OWA: Outer wall area
PCA: Posterior cerebral artery
PICA: Posterior inferior cerebellar artery
RI: Remodeling index
TIA: Transient ischemic attack
TOAST: Trial of ORG 10172 in acute stroke Treatment
VA: Vertebral artery
WASID: Warfarin-aspirin symptomatic intracranial disease

## References

[1] Savitz SI, Caplan LR (2005). Vertebrobasilar disease. N Engl J Med.

[2] Gulli G, Marquardt L, Rothwell PM, Markus HS (2013). Stroke risk after posterior circulation stroke/transient ischemic attack and its relationship to site of vertebrobasilar stenosis: pooled data analysis from prospective studies. Stroke..

[3] Jeng JS, Yip PK (2004). Evaluation of vertebral artery hypoplasia and asymmetry by color-coded duplex ultrasonography. Ultrasound Med Biol.

[4] Perren F, Poglia D, Landis T, Sztajzel R (2007). Vertebral artery hypoplasia: a predisposing factor for posterior circulation stroke?. Neurology..

[5] Hong JM, Chung CS, Bang OY, Yong SW, Joo IS, Huh K (2009). Vertebral artery dominance contributes to basilar artery curvature and peri-vertebrobasilar junctional infarcts. J Neurol Neurosurg Psychiatry.

[6] Lochner P, Golaszewski S, Caleri F, Ladurner G, Tezzon F, Zuccoli G (2011). Posterior circulation ischemia in patients with fetal-type circle of Willis and hypoplastic vertebrobasilar system. Neurol Sci.

[7] Arjal RK, Zhu T, Zhou Y (2014). The study of fetal-type posterior cerebral circulation on multislice CT angiography and its influence on cerebral ischemic strokes. Clin Imaging.

[8] Holmstedt Christine A, Turan Tanya N, Chimowitz Marc I (2013). Atherosclerotic intracranial arterial stenosis: risk factors, diagnosis, and treatment. The Lancet Neurology.

[9] Abuzinadah AR, Alanazy MH, Almekhlafi MA, Duan Y, Zhu H, Mazighi M (2016). Stroke recurrence rates among patients with symptomatic intracranial vertebrobasilar stenoses: systematic review and meta-analysis. J Neurointerv Surg.

[10] Lou X, Ma N, Ma L, Jiang WJ (2013). Contrast-enhanced 3T high-resolution MR imaging in symptomatic atherosclerotic basilar artery stenosis. AJNR Am J Neuroradiol.

[11] Amin-Hanjani S, Pandey DK, Rose-Finnell L, Du X, Richardson D, Thulborn KR (2016). Vertebrobasilar flow evaluation and risk of transient ischemic attack and stroke study group. Effect of hemodynamics on stroke risk in symptomatic atherosclerotic Vertebrobasilar occlusive disease. JAMA Neurol.

[12] Alves HC, Treurniet KM, Dutra BG, Jansen IGH, Boers AMM, Santos EMM (2018). MR CLEAN trial investigators. Associations between collateral status and Thrombus characteristics and their impact in anterior circulation stroke. Stroke..

[13] Tan IY, Demchuk AM, Hopyan J, Zhang L, Gladstone D, Wong K (2009). CT angiography clot burden score and collateral score: correlation with clinical and radiologic outcomes in acute middle cerebral artery infarct. AJNR Am J Neuroradiol.

[14] Ay H, Furie KL, Singhal A, Smith WS, Sorensen AG, Koroshetz WJ (2005). An evidence-based causative classification system for acute ischemic stroke. Ann Neurol.

[15] Qiao Y, Zeiler SR, Mirbagheri S, Leigh R, Urrutia V, Wityk R (2014). Intracranial plaque enhancement in patients with cerebrovascular events on high-spatial-resolution MR images. Radiology..

[16] Qiao Y, Anwar Z, Intrapiromkul J, Liu L, Zeiler SR, Leigh R, Zhang Y (2016). Patterns and implications of intracranial arterial remodeling in stroke patients. Stroke..

[17] Ma N, Jiang WJ, Lou X, Ma L, Du B, Cai JF, Zhao TQ (2010). Arterial remodeling of advanced basilar atherosclerosis: a 3-tesla MRI study. Neurology..

[18] Chimowitz MI, Kokkinos J, Strong J, Brown MB, Levine SR, Silliman S (1995). The warfarin-aspirin symptomatic intracranial disease study. Neurology..

[19] Chen Z, Liu AF, Chen H, Yuan C, He L, Zhu Y (2016). Evaluation of basilar artery atherosclerotic plaque distribution by 3D MR vessel wall imaging. J Magn Reson Imaging.

[20] Yu JH, Kwak HS, Chung GH, Hwang SB, Park MS, Park SH (2015). Association of intraplaque hemorrhage and acute infarction in patients with basilar artery plaque. Stroke..

[21] Ma N, Xu Z, Lyu J, Li M, Hou Z, Liu Y (2019). Association of Perforator Stroke after Basilar Artery Stenting with Negative Remodeling. Stroke..

[22] van Raamt AF, Mali WP, van Laar PJ, van der Graaf Y (2006). The fetal variant of the circle of Willis and its influence on the cerebral collateral circulation. Cerebrovasc Dis.

[23] Ravensbergen J, Ravensbergen JW, Krijger JK, Hillen B, Hoogstraten HW (1998). Localizing role of hemodynamics in atherosclerosis in several human vertebrobasilar junction geometries. Arterioscler Thromb Vasc Biol.

[24] Bodle JD, Feldmann E, Swartz RH, Rumboldt Z, Brown T, Turan TN (2013). High-resolution magnetic resonance imaging: an emerging tool for evaluating intracranial arterial disease. Stroke..

[25] Millon A, Boussel L, Brevet M, Mathevet JL, Canet-Soulas E, Mory C (2012). Clinical and histological significance of gadolinium enhancement in carotid atherosclerotic plaque. Stroke..

[26] Xu WH, Li ML, Gao S, Ni J, Yao M, Zhou LX (2012). Middle cerebral artery intraplaque hemorrhage: prevalence and clinical relevance. Ann Neurol.

[27] Smith AS, Bellon JR (1995). Parallel and spiral flow patterns of vertebral artery contributions to the basilar artery. AJNR Am J Neuroradiol.

[28] Dolan JM, Kolega J, Meng H (2013). High wall shear stress and spatial gradients in vascular pathology: a review. Ann Biomed Eng.

[29] Suh DC, Park ST, Oh TS, Park SO, Lim OK, Park S (2011). High shear stress at the surface of enhancing plaque in the systolic phase is related to the symptom presentation of severe M1 stenosis. Korean J Radiol.

[30] Tuenter A, Selwaness M, Arias Lorza A, Schuurbiers JCH, Speelman L, Cibis M (2016). High shear stress relates to intraplaque haemorrhage in asymptomatic carotid plaques. Atherosclerosis..

[31] Han HC (2012). Twisted blood vessels: symptoms, etiology and biomechanical mechanisms. J Vasc Res.

[32] Kim BJ, Lee KM, Kim HY, Kim YS, Koh SH, Heo SH (2018). Basilar artery plaque and Pontine infarction location and vascular geometry. J Stroke.

