# Supplementary material for: Association between basilar artery configuration and Vessel Wall features: a prospective high-resolution magnetic resonance imaging study
Source: BMC Med Imaging. 2019 Dec 26;19:99. doi: 10.1186/s12880-019-0388-3 (PMC6933671; doi:10.1186/s12880-019-0388-3)
Supplement: Supplementary file 1 — Additional file 1: Tables S1. Parameters of multiple sequences on GE and Siemens MR scanners. [file 12880_2019_388_MOESM1_ESM.docx]

| Table S1. Parameters of multiple sequences on GE and Siemens MR scanners. | | | | | | | | |
| --- | --- | --- | --- | --- | --- | --- | --- | --- |
|  |  |  | **GE** |  |  |  | **Siemens** |  |
|  | 3D TOF | 3D CUBE T1 | 3D CUBE PD | MP RAGE | 3D TOF | 3D SPACE T1 | 3D SPACE PD | MP RAGE |
| TR, ms | Minimum | 600 | 1500 | Minimum | 24 | 800 | 1700 | 776.13 |
| TE, ms | Minimum | Minimum | 40 | 3.3 | 4.32 | 22 | 23 | 5.8 |
| FOV, mm^2^ | 240×220 | 240×220 | 240×220 | 240×240 | 140×140 | 180×168 | 180×180 | 144×144 |
| Matrix | 512×512 | 480×480 | 384×384 | 256×256 | 256×256 | 256×251 | 320×304 | 240×240 |
| Slice thickness, mm | 2 | 0.8 | 0.8 | 0.8 | 0.9 | 0.8 | 0.6 | 1 |
| Flip angle, degree | 20 | 90 | 90 | 12 | 18 | 120 | 120 | 15 |
| Scan time, min | 1:53 | 3:33 | 2:40 | 2:18 | 2:14 | 4:40 | 4:25 | 2:39 |

Abbreviations:3D,3 dimensional; T1, T1-weighted imaging; PD, proton weighted imaging; MPRAGE, magnetization-prepared rapid acquisition with gradient-echo sequence; TR, repetition time; TE, echo time; FOV, field of view; TOF, time of flight.
